# Bio-Scaffolds as Cell or Exosome Carriers for Nerve Injury Repair

**DOI:** 10.3390/ijms222413347

**Published:** 2021-12-12

**Authors:** Raju Poongodi, Ying-Lun Chen, Tao-Hsiang Yang, Ya-Hsien Huang, Kuender D. Yang, Hsin-Chieh Lin, Jen-Kun Cheng

**Affiliations:** 1 Department of Medical Research, Mackay Memorial Hospital, Taipei 10449, Taiwan; poongodiraju@gmail.com (R.P.); konnankonnan@yahoo.com.tw (T.-H.Y.); 2 Department of Anesthesiology, Mackay Memorial Hospital, Taipei 10449, Taiwan; alexingchaning@gmail.com (Y.-L.C.); trista468@gmail.com (Y.-H.H.); 3 Department of Medicine, Mackay Medical College, New Taipei City 25245, Taiwan; 4 Institute of Biomedical Science, Mackay Medical College, New Taipei City 25245, Taiwan; yangkd.yeh@hotmail.com; 5 Department of Pediatrics, Mackay Memorial Hospital, Taipei 10449, Taiwan; 6 Institute of Clinical Medicine, National Yang Ming Chiao Tung University, Taipei 11221, Taiwan; 7 Department of Materials Science and Engineering, National Yang Ming Chiao Tung University, Hsinchu 30010, Taiwan; hclin45@nctu.edu.tw

**Keywords:** bio-scaffold, biomaterial, exosome, motor function, natural polymer, nerve injury, nerve regeneration

## Abstract

Central and peripheral nerve injuries can lead to permanent paralysis and organ dysfunction. In recent years, many cell and exosome implantation techniques have been developed in an attempt to restore function after nerve injury with promising but generally unsatisfactory clinical results. Clinical outcome may be enhanced by bio-scaffolds specifically fabricated to provide the appropriate three-dimensional (3D) conduit, growth-permissive substrate, and trophic factor support required for cell survival and regeneration. In rodents, these scaffolds have been shown to promote axonal regrowth and restore limb motor function following experimental spinal cord or sciatic nerve injury. Combining the appropriate cell/exosome and scaffold type may thus achieve tissue repair and regeneration with safety and efficacy sufficient for routine clinical application. In this review, we describe the efficacies of bio-scaffolds composed of various natural polysaccharides (alginate, chitin, chitosan, and hyaluronic acid), protein polymers (gelatin, collagen, silk fibroin, fibrin, and keratin), and self-assembling peptides for repair of nerve injury. In addition, we review the capacities of these constructs for supporting in vitro cell-adhesion, mechano-transduction, proliferation, and differentiation as well as the in vivo properties critical for a successful clinical outcome, including controlled degradation and re-absorption. Finally, we describe recent advances in 3D bio-printing for nerve regeneration.

## 1. Introduction

Tissue engineering combines findings from cell biology and material science to mimic the physical and chemical conditions of native tissue with the aim of functional restoration following injury [1]. The major focus of modern tissue engineering is repair and regeneration of the central nervous system (CNS) and peripheral nervous system (PNS) as these tissues have limited inherent regenerative potential in mammals [2], but numerous challenges remain before routine clinical application. Among the most pressing of these challenges is the fabrication of three-dimensional (3D) scaffolds able to sustain the survival and guide the proliferation, functional differentiation, and targeting of transplanted replacement or supporting cells.

**Figure 1:**
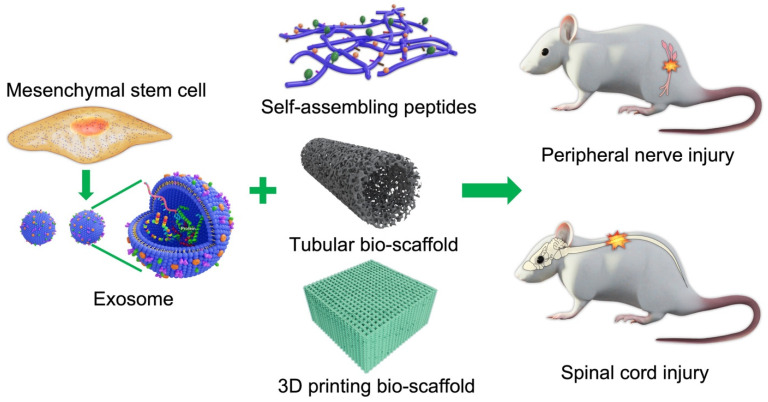
Schematic illustration of cell/exosome and bio-scaffold combinations for the treatment of central and peripheral nerve injury.

Scaffolds are well-known 3D porous functional biomaterials possessing constructive characteristics such as offering the proper position of cell location, cell adhesion, and deposition of the extracellular matrix (ECM) [3]. Moreover, scaffolds allow adequate gas transport, essential nutrients, and controlling factors to promote cell proliferation, survival, and differentiation. Based on their origin, scaffolds can be broadly classified/differentiated into natural/biological (such as collagen, chitosan, glycosaminoglycans, hyaluronic acid, demineralized, or native dentin matrix, etc.) and synthetic (such as bio-ceramics, calcium phosphate, and bioactive glasses, etc.) [3]. Biopolymer-based scaffolds are useful materials for 2D and 3D cell culture [4] and drug loading [5], and have demonstrated some value for tissue regeneration in various preclinical models [6,7]. Ideal scaffolds must possess the ability to replace damaged tissues with exogenous (transplanted) or endogenous cells of the correct tissue architecture for functional restoration [8]. For example, nerve damage is common following limb or head trauma and is frequently irreversible or difficult to treat [9]. One major reason for this irreversibility is the absence of a growth-permissive environment following injury, so biocompatible scaffold materials are needed to enhance repair [10,11]. In addition to high biocompatibility [12], scaffold materials should also have tunable mechanical strength [13], a large surface area, high porosity [14], and surface properties that mimic the physical and chemical properties of the ECM [15] and lack potential biotoxicity [16] in order to promote cell-adhesion, proliferation, and differentiation [17]. The appropriate chemical environment may be provided by biomaterials that can be loaded with cells or exosomes supplying nutritive and trophic factors to the injury site (illustrated in Figure 1). Herein, we describe the use of various bio-scaffolds for treating nerve injury.

## 2. Mesenchymal Stem Cells for Tissue Replacement

Mesenchymal stem cells (MSCs) are multipotent progenitors present in the skin, dental pulp, adipose tissue, bone marrow, and umbilical cord with the capacity to differentiate into hepatocytes, chondrocytes, osteoblasts, adipocytes, cardiomyocytes, neurons, and glial cells among other cell types under specific conditions [18]. For instance, MSCs are readily differentiated into neurons by culturing in media or CSF previously incubated with fetal or neonatal brain tissue (conditioned media) to supply the appropriate neurotropic factors [19]. Further, MSCs can also be successfully differentiated into glial cells in situ [20]. In addition to chemical properties, the mechanical properties of the microenvironment (scaffold) also influence the differentiation pathway of MSCs [21].

## 3. Exosomes

A major challenge for regeneration and functional restoration is to supply the various nutritive and growth factors required for cell survival, growth, and differentiation. Exosomes are biological nanoscale (30–120 nm in diameter) lipid bilayer vesicles secreted by cells. According to the 2021 ExoCarta database, around 4946 RNAs, 41,860 proteins, and 1116 lipids as well as various DNA sequences, mRNAs, and non-coding RNAs have been detected in various exosomes [22]. MicroRNAs are among the most enriched of the microsomal non-coding RNA species and have been implicated in the local angiogenesis, exocytosis, hematopoiesis, and cell–cell communication mediated by these vesicles [23]. Other exosomal RNA species include transfer RNAs, long non-coding RNAs, ribosomal RNAs, and both small nuclear and nucleolar RNAs [24]. Exosomes also express surface proteins such as CD81, CD9, CD63, and TSG101 that allow these structures to bind and transport contents into target cells and thus regulate specific biological functions such as neurotransmission, intercellular signaling, angiogenesis, tumor cell proliferation, metastasis, and immune responses [25,26]. Compared to MSCs for scaffold loading, exosomes are easier to store, less tumorigenic, and less likely to be reprogrammed by environmental factors [27]. For regenerative medicine, Codispoti et al. have proposed the development of the NANOmetric BIO-banked MSC-derived Exosome (NANOBIOME) to be used in different timepoints and disease models [28].

## 4. Natural Polymeric Scaffolds

Natural polymeric bio-scaffolds are fabricated with structural components and chemical signaling molecules that stimulate cell survival, proliferation, differentiation, and tissue reconstruction, such as neurotrophic factors and vascular endothelial growth factor (VEGF). The correct combination of factors and appropriate bioavailability is required for nerve regeneration after injury. Natural polymers used as structural components include various polysaccharides such as alginate, hyaluronic acid, chitin, and chitosan, and polymeric proteins such as gelatin, collagen, silk fibroin, fibrin, and keratin [29,30]. All of the polymers have excellent biocompatibility and bioactive properties and so may allow for better scaffold–tissue interactions as well as cell adhesion, proliferation, and eventual tissue restoration [31]. However, some lack the biophysical characteristics for functional recovery. The basic properties and various advantages and disadvantages of these compounds for bio-scaffolds are described below.

### 4.1. Polysaccharide-Based Biomaterials

#### 4.1.1. Hyaluronic Acid

Hyaluronic acid (HA) is a glycosaminoglycan component of ECM that facilitates the interactions of cells with other extracellular molecules to promote various physiological processes [32]. Further, HA in the ECM has been implicated in angiogenesis, tumorigenesis, inflammatory processes, drug resistance, water homeostasis, and regulation of viscoelasticity [33]. Using microbial technology, HA can be obtained in large amounts without the risk of contamination by animal pathogens. In addition, the extent of HA degradation can be modified by crosslinking with divinyl sulfone. This crosslinking also creates a porous structure after freezing and lyophilization that provides additional surface area for cell proliferation [33,34]. Alternatively, HA can be resolved with sodium chloride and directly poured into a porous sponge [35,36]. Non-adhesive and biocompatible HA can support axonal regeneration, but is structurally too weak for most human regenerative applications unless combined with other natural materials such as chitosan [35].

HA has been used successfully with different substrates to support neurite out-growth, differentiation, and proliferation. Further, HA hydrogel has been used to promote the survival and proliferation of neural precursors for PNS repair [37] and has shown promise for CNS repair. It has mechanical properties suitable for supporting neural progenitor cell differentiation as potential neurodegenerative disease treatments [38]. Long-chain HA is essential for supporting ECM components of different molecular weights in vivo [39]. An HA scaffold containing ciliary neurotrophic factor stimulated endogenous neurogenesis and facilitated neural-network formation, synaptogenesis, and motor recovery following T8 spinal cord transection in rodents [40].

#### 4.1.2. Alginate

Alginate, an extract of brown seaweed, is used for a variety of biomedical applications. Its chemical composition of guluronic and mannuronic acid confers greater chemical flexibility compared to other biocompatible degradable materials and may more closely mimic the physical properties of mammalian ECM [41]. Physical and mechanical properties are also easily adjustable using various chemical reactions [42] and physical crosslinking using Ca^2+^ with negligible immunogenicity [43]. While alginate can promote nerve regeneration under certain conditions, mechanical strength is insufficient to allow physical loading, and degradation is relatively rapid, necessitating the addition of other polymers [44,45,46]. For example, alginate hydrogel covalently cross-linked with *N,N′*-disuccinimidyl carbonate has been combined with electrospun polycaprolactone nanofibers to produce a bilayer cylindrical conduit for sciatic nerve repair [47]. In our previous study, we also used an alginate scaffold as a stem cell exosome carrier for the treatment of nerve injury-induced pain [48].

#### 4.1.3. Chitosan and Chitin

Chitin is the most abundant linear polysaccharide homo-polymer of the glycosaminoglycan *N*-acetyl-D-glucosamine in crustacean shells. In fact, half of shellfish waste consists of chitin derivatives, and these can be extracted by microbiological or chemical methods [49]. Chitosan-silk hydrogel as a carrier for gingival MSC-derived exosomes was reported to accelerate neurogenesis, angiogenesis, re-epithelization, and collagen formation [50]. In a mouse hind-limb repair model as well, animals receiving MSC exosomes encapsulated with chitosan exhibited better angiogenesis and tissue regeneration than controls [51].

Chitosan is also commonly used to support axon regrowth [52] and reduce scar tissue formation [53] for peripheral nerve regeneration. Further, both reabsorbing chitosan and its degradation products (chito-oligosaccharides) have been shown to promote nerve regeneration [54]. Using appropriate fabrication techniques, chitosan nerve guidance conduits can be produced for cell-based therapies [55,56]. In many studies of rat transection models, chitosan tubes have improved nerve regeneration by linking the defective peripheral nerve ends [57,58,59,60]. After sciatic nerve injury, chitosan nanoparticles with encapsulated neural growth factor also promoted Schwann cell proliferation and nerve regeneration [61], while another study found the animals receiving chitosan embedded nerve implants showed more numerous axons than control [62]. Chitosan can provide a permissive surface for nerve regeneration, and then degrade without inducing inflammation. For instance, a chitosan catheter induced significant sensory and motor axon regeneration after long distance transection [62]. In another nerve defect model, animals receiving stem cells embedded in a chitosan scaffold showed target muscle re-innervation [63]. Recently, Bo et al. reported that chitin scaffolds with autologous nerve tissue promoted sciatic nerve regeneration, myelin sheath formation, and neurological recovery [64].

## 5. Protein-Based Biomaterials for Nerve Injuries

### 5.1. Collagen

Collagen is a highly flexible natural polymeric protein and the major protein component of the ECM. Endogenous collagen contributes to the maintenance of ECM structural integrity and spatial organization and thus is essential for ECM deposition as well as natural tissue morphogenesis, repair, and re-modeling. Further, de-cellularized collagen matrices can be separated and treated with immunogenic antigens while retaining the original ECM organization of functional proteins.

Collagen scaffolds have numerous advantages for tissue engineering [65]. Collagen is a good medium for cell and drug delivery [66] and is sufficiently flexible for nerve conduits with physical features tailored for different sections of the nerve pathway [67]. In addition, it can support topographical cues that allow axonal regrowth and facilitate cell-adhesion, survival, and migration along different nerve tract domains [68]. Such collagen nerve conduits have been demonstrated to support nerve regeneration and re-innervation of muscle [69]. In a clinical study, a conduit made by mixing type I and III collagen filled with collagen filaments was effective as an autologous implant for treating nerve injury, with 75% of patients reporting sensory recovery after 12 months [70]. A collagen scaffold embedded with neural stem cells was also reported to promote nerve regeneration and motor function in a T8 SCI rat model [71]. Further, the scaffold was completely gone at 5 weeks after implantation, indicating good biodegradability. In a traumatic brain injury model, a collagen–heparin scaffold with VEGF stimulated angiogenesis and promoted nerve regeneration, likely due to its excellent mechanical properties, good porosity, and control of VEGF release [72]. Even though there is always a concern for the immunogenicity of collagen, most of the literature dealing with immunochemistry of collagen-based materials indicate that a proper research investigation is necessary to ensure the outcomes derived from a specific donor or recipient should not be applied to make extensive generalizations with respect to the immunological compatibility of various collagen types [73].

### 5.2. Laminin

Laminins are high molecular weight proteins that constitute the major component of the ECM basal lamina layer, a protein network that acts as a structural foundation for most organs and cells. Laminin proteins are also a major component of the brain ECM and function as cell adhesion molecules influencing cell survival, differentiation, and plasticity. For instance, laminins were shown to promote the survival and differentiation of transplanted dopaminergic neuron precursors by suppressing cell death-associated protein [74]. Additionally, laminin present in the vascular basal lamina can act as a conduit for the growth of axons [75] as it is expressed endogenously in the basal membrane surrounding peripheral nerves, capillaries, and skeletal muscle. Further, it can regulate the proliferation, differentiation, and myelin production of Schwann cells. Laminins are also secreted by Schwann cells at lesion sites [76], strongly suggesting functions in nerve repair. For these reasons, laminins are considered promising scaffold components for nerve repair [77,78]. Indeed, nerve guides filled with laminin yielded enhanced axonal regeneration [79], likely by increasing the interactions with integrin receptors.

### 5.3. Gelatin

Denatured collagen can be converted to gelatin by high temperature or treatment with strong acid, base or enzyme [80]. Dissolved in water, gelatin is a biocompatible and biodegradable polymer that forms a hydrogel with thermo-sensitive holding properties. Further, the occurrence of an arginyl-glycyl-aspartic acid (RGD) sequence and integrin-binding molecules in gelatin material has promoted cell attachment and multiplying [81]. On the other hand, gelatin-based hydrogels may have low viscosity at physiological temperature, limiting the maintenance of the 3D structure. To increase its strength, gelatin is combined with other polymers, such as collagen, fibrin, or various synthetic and photo-crosslinkable polymers [82,83]. Though different kinds of gelatin-based hydrogels such as micro- and nano-sized particles, nanofibrous scaffolds, enzyme-mediated, and in situ-generated gelatin hydrogels were reported [84]; the enzymatically prepared gelatin hydrogels have been widely used in nerve regeneration. For instance, the enzymatically prepared gelatin hydrogels combined with human umbilical cord MSCs have been effectively applied for nerve injury treatment [85,86].

### 5.4. Silk Fibroin

Silk fibroin (SF) is a natural biopolymer with high biocompatibility [87] and low immunogenicity [88] as well as sufficient biodegradability [89], physical strength, and flexibility for in vivo applications [90]. SF has been shown to promote cell attachment and survival for tissue repair and restoration [91]. Further, SF can promote proliferation of Schwann cells [92] and so may be especially effective for peripheral nerve regeneration. In addition, an SF-based hydrogel was also demonstrated to support neuronal growth for central nerve tissue repair [93]. Critically, the orientation of SF fibers can guide the direction of neuronal growth [94]. These unique properties may explain the efficacy of SF fibers for promoting neural cell proliferation following auto- or allo-grafting [95]. In addition, SF can deliver bioactive compounds to the injury site and reduce both tissue inflammation and oxidative stress. Moreover, SF fibers show slow biodegradation [96]. In a traumatic brain injury model, SF reduced brain damage and promoted neurological function [97].

**Figure 2:**
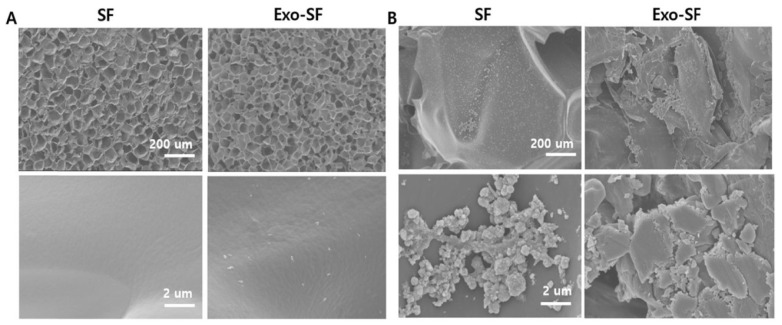
In vitro studies of silk fibroin (SF) and stem cell exosome (Exo)-embedded scaffolds. (**A**) Scanning electron microscope images of bare SF and Exo-SF scaffold surfaces. (**B**) Morphology of human bone marrow-derived mesenchymal stem cells cultured on SF and Exo-SF scaffolds for 2 weeks. Reprinted with permission from Kyung Kim, D.; Lee, S.; Kim, M.; Jeong, Y.; Lee, S. (2021). Copyright 2021 Chemical Engineering Journal, Elsevier [98].

SF scaffolds can be synthesized in various conformations such as fibers, mats, films, and hydrogels. This adaptability may permit its application for the treatment of several neurogenerative diseases in addition to traumatic nerve injury. Due to its unique physico-chemical and biological properties, SF is a promising material for tissue engineering. Recently, SF 3D-scaffolds enriched in MSC-derived exosomes were also reported to enhance bone regeneration in rats [98] (Figure 2).

### 5.5. Fibrin

Fibrin is a fibrillary protein formed during blood clotting. It is mainly involved in hemostasis, but also contributes to wound healing by forming a temporary matrix surrounding the lesion [99]. Changes in the fibrinogen-to-thrombin ratio can modulate the mechanical properties of fibrin hydrogels for effective treatment of human spinal cord injury [100]. Due to its high biocompatibility, fibrin has been used as a vehicle and injectable biomaterial for transplantation of cells to facilitate neural regeneration [101]. The mechanical properties of fibrin hydrogels are also highly tunable by altering the fibrin concentration and preparation temperature [102]. Unfortunately, fibrin conduits cannot be sutured due to low mechanical elasticity, and a suture-less conduit may be unable to maintain a cohesive nerve structure [103]. Nevertheless, fibrin conduits with fibrin matrix or human MSCs can be used to promote axonal regeneration and reduce muscle atrophy after sciatic nerve injury [104]. In an SCI model, a 3D fibrin scaffold provided an effective matrix for host cell invasion and vascular reconstruction, thereby promoting axonal regrowth and recovery of locomotor function [105]. Following sciatic nerve injury, a Wnt5a-loaded fibrin conduit was also reported to promote neurotrophin secretion and nerve regeneration [106].

### 5.6. Keratin

**Figure 3:**
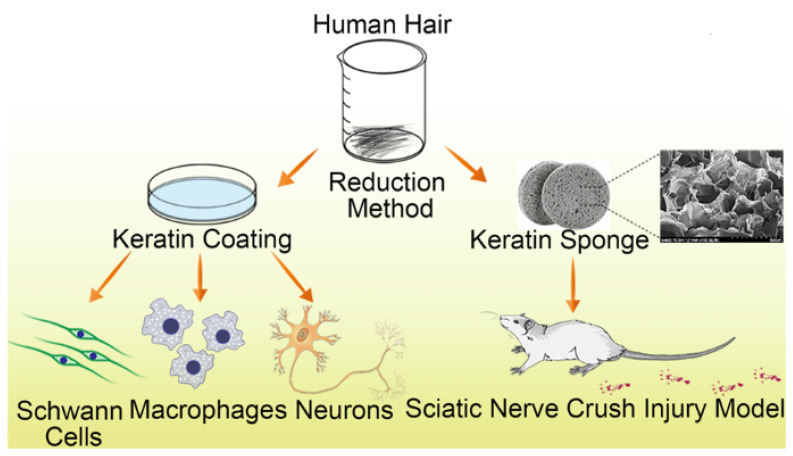
Schematic illustration of keratin sponge application for nerve regeneration following sciatic nerve crush injury. Keratin biomaterial promoted Schwann cell proliferation and regulated macrophage inflammatory cytokines and elongation of the axon in dorsal root ganglion neurons in vitro. Likewise, in vivo studies demonstrated that keratin sponge restored motor function after sciatic nerve crush injury. Reprinted with permission from Gao, J.; Zhang, L.; Wei, Y.; Chen, T.; Ji, X.; Ye, K.; Yu, J.; Tang, B.; Sun, X.; Hu, J. (2019). Copyright 2019 Journal of Materials Science: Materials in Medicine, Springer Nature [109].

**Figure 4:**
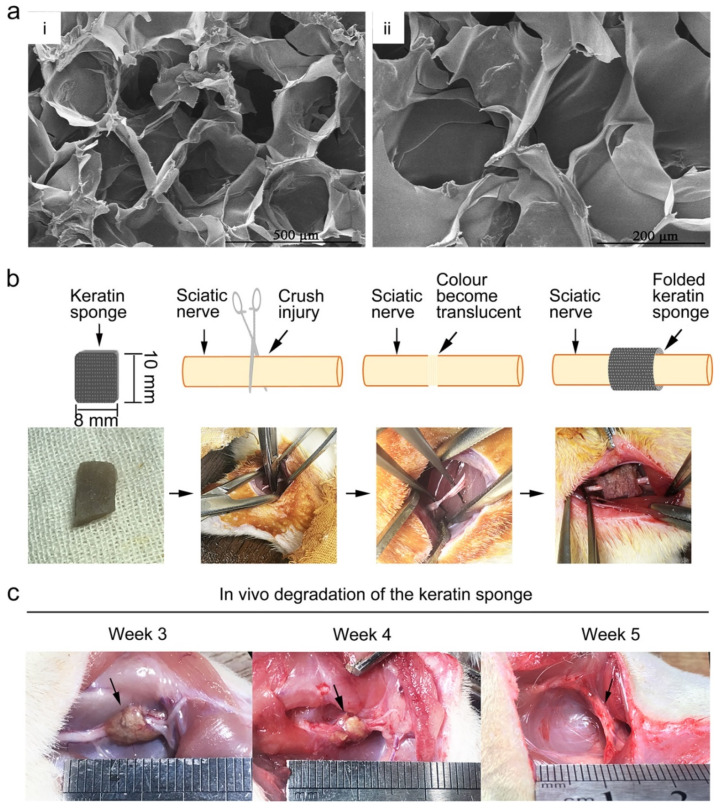
Sciatic nerve injury repair using keratin sponge. (**a**) Scanning electron microscope showing the micro-porous structure of keratin sponge. (**b**) Schematic illustration of the sciatic nerve crush injury model. (**c**) Keratin sponge degradation. Reprinted with permission from Gao, J.; Zhang, L.; Wei, Y.; Chen, T.; Ji, X.; Ye, K.; Yu, J.; Tang, B.; Sun, X.; Hu, J. (2019). Copyright 2019 Journal of Materials Science: Materials in Medicine, Springer Nature [109].

Keratin can be extracted from human hair and further processed to obtain a keratin sponge structure. Compared to many synthetic polymers, keratin appears to possess the surface hydrophilicity, biodegradability, biocompatibility, and bioactivity of an effective scaffold material. However, keratin-based biomaterials have low mechanical strength and degrade rapidly, and so are usually modified using various crosslinking agents for scaffold construction [107], while keratin alone is used primarily as a conduit filler. Keratin/alginate scaffolds have been applied successfully for tissue regeneration in vitro [108]. Furthermore, keratin has been shown to promote Schwann cell proliferation in vitro and improve nerve regeneration in vivo [109,110] (Figure 3 and Figure 4).

## 6. Self-Assembling Peptides

Self-assembling peptides (SAPs) can spontaneously form well-organized nanostructures, a property highly advantageous for a wide range of biomedical applications. For nerve injuries, SAPs have been used as biocompatible carriers to provide the appropriate 3D structure for embedded nerve cells and the release of growth factors and drugs [111]. Moreover, SAPs have been shown to provide a microenvironment conducive to cell proliferation and differentiation as well as neural-network reconstruction and functional restoration of injured nerves [112,113,114].

SAPs may be ideal building blocks for scaffolds and can also be used as soft fillers to surround harder synthetic biocompatible biopolymers. In general, the scaffold must imitate the natural biomechanical properties of the regenerating tissue and permit the cell–substrate and cell–cell interactions necessary for regrowth. Further, bio-absorption must be appropriately matched to tissue regeneration kinetics and result in little inflammation [115]. Many clinical studies have attempted to produce SAP-based scaffolds with these properties and examined the efficacy for regeneration.

**Figure 5:**
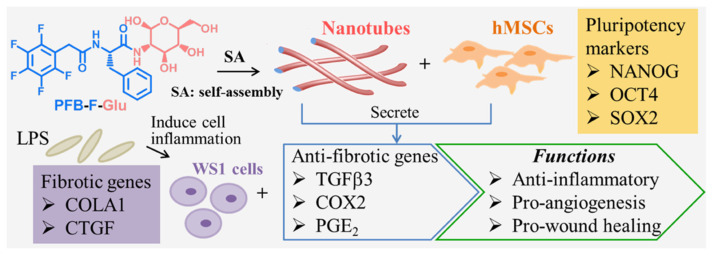
Glucosamine-based supramolecular nanotube formation and research strategy for stem cell therapy. PFB-F-Glu (pentafluorobenzyl-phenylalanine-glucosamine) nanotubes are shown to enhance hMSC (human mesenchymal stem cells) proliferation while maintaining their pluripotency. The hMSCs cultured on PFB-F-Glu nanotubes could secrete paracrine factors to suppress pro-fibrotic gene expression in lipopolysaccharide (LPS)-treated human skin fibroblasts (WS1), indicating the nanotubes have the potential for wound healing treatment. OCT4: octamer-binding transcription factor 4; SOX2: SRY (sex determining region Y)-box 2; COLA1: collagen α1 chain; CTGF: connective tissue growth factor; TGFβ3: transforming growth factor β3; COX2: cyclooxygenase 2; PGE_2_: prostaglandin E_2_. Reprinted with permission from Talloj, S.K.; Cheng, B.; Weng, J.-P.; Lin, H.-C. (2018). Copyright 2018 ACS Applied Materials & Interfaces, American Chemical Society [121].

The most common peptide sequences used for self-assembly are RGD, IKVAV (isoleucine-lysine-valine-alanine-valine), YIGSR (tyrosine-isoleucine-glycine-serine-arginine), and RADA16 (4 arginine-alanine-aspartate-alanine repeats or RADARADARADARADA). RADA16 balances lipophilic and hydrophilic peptide interactions [116], while IKVAV can promote cell differentiation, adhesion, and axon growth in injured nerves [117]. Further, Zhang et al. have introduced these two efficient SAP sequences, IKVAV and RADA16-I, into self-assembled nanofiber hydrogels to enhance the axon extension, cell attachment, and neuroregeneration [46,118,119,120]. Similarly, Talloj et al. synthesized a series of amino acid derivatives by capping D-glucosamine at the C-terminus and fluorinated benzyl group at the N-terminus. They found the glucosamine-based supramolecular hydrogel (pentafluorobenzyl (PFB)-F-Glu) could self-assemble into nanotubules, which can increase human MSC proliferation and the secretion of paracrine factors that downregulate pro-fibrotic gene expression of human skin fibroblasts [121] (Figure 5).

## 7. Three-Dimensional Printed Scaffolds

**Figure 6:**
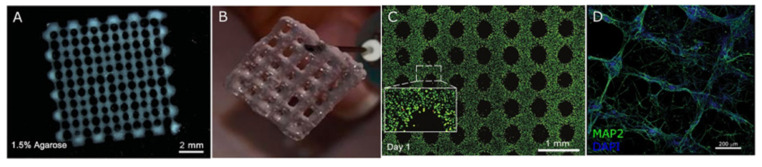
Images of various 3D bio-printed scaffolds. (**A**) Human neural stem cells (hNSCs) embedded in an alginate (Al)/agarose (Ag)/carboxymethyl-chitosan (CMC)-based hydrogel. Reprinted with permission from Gu, Q.; Tomaskovic-Crook, E.; Lozano, R.; Chen, Y.; Kapsa, R.M.; Zhou, Q.; Wallace, G.G.; Crook, J.M. (2016). Copyright 2016 Advanced Healthcare Materials, John Wiley and Sons [122]. (**B**) Neuroblastoma cells embedded in Al/gelatin hydrogel. Reprinted with permission from Fantini, V.; Bordoni, M.; Scocozza, F.; Conti, M.; Scarian, E.; Carelli, S.; Di Giulio, A.M.; Marconi, S.; Pansarasa, O.; Auricchio, F.; et al. (2019). Copyright 2019 Cells, MDPI [126]. (**C**) Live–dead (green/red) cell staining of an Al/Ag/CMC-based hydrogel containing induced pluripotent stem cells (iPSCs). Reprinted with permission from Gu, Q.; Tomaskovic-Crook, E.; Wallace, G.G.; Crook, J.M. (2017). Copyright 2017 Advanced Healthcare Materials, John Wiley and Sons [127]. (**D**) Neuronal alignment within a Matrigel/Al hydrogel. MAP2: microtubule-associated protein 2. Reprinted with permission from Salaris, F.; Colosi, C.; Brighi, C.; Soloperto, A.; de Turris, V.; Benedetti, M.C.; Ghirga, S.; Rosito, M.; Di Angelantonio, S.; Rosa, A. (2019). Copyright 2019 Journal of Clinical Medicine, MDPI [128].

Three-dimensional (3D) bio-printing is used extensively in regenerative medicine, cancer research, and the pharmaceutical industry to fabricate structures combining cells, growth factors, and cell substrates. Three-dimensional printed scaffolds have been demonstrated to stimulate cell attachment, growth, and organization resembling nervous tissue. In addition, 3D bio-printing has been used to create scaffolds with defined porosity and inter-pore channel structure. Currently, two modes of 3D printing are used to create 3D cell-embedded scaffolds and scaffolds with supportive bio-ink. Both types can help to reconstruct the cellular structure of the original tissue. Bio-ink printing can quickly form porous 3D scaffolds encapsulating human neural stem cells able to differentiate and replace lost function and/or support the growth of other neurons and glia [122]. For example, Bociaga et al. demonstrated that bio-printing can produce scaffolds with excellent microstructural features for cell growth [123]. Moreover, these fabrication techniques have shown promise for printing tissue components such as grafts and organs. One recent study reported the development of a microsphere-loaded bio-ink to print scaffolds with neural progenitor cells (NPCs) for neural tissue repair [124], and another reported promising results using printed scaffolds for regeneration following sciatic nerve injury [125]. Some of these 3D bio-printed biomaterials are illustrated in Figure 6. In addition, recent examples of bio-scaffold applications for in vitro and in vivo nerve injury repair are summarized in Table 1 and Table 2.

## 8. Bio-Scaffolds for Exosomes

Several recent studies have also described the fabrication and utility of bio-scaffolds for exosomes. These bio-scaffold should have following advantages: (1) they can efficiently maintain the exosomes at the injury site and retain their performance and structural characteristics; (2) they release exosomes into the ECM for a sufficient period to adjust the phenotype of neighboring cells; (3) they can integrate with injured tissue to support neighboring cell migration into the scaffold. Once the neighboring cells migrate into the bio-scaffold, the exosomes can be absorbed and enhance tissue regeneration. For exosome loading, physical implanting and diffusion are the two widely reported methods. The dispersion of exosomes mainly depends on the porosity and cross-linking density of the bio-scaffold.

Many studies were performed to assemble ionic cross-linking bio-scaffolds for exosome maintenance and release. In this regard, alginate hydrogel is considered one of the best bio-scaffold for encapsulating exosomes. For instance, an exosome-loaded alginate scaffold has been reported to improve collagen production, skin regeneration, and angiogenesis in the wound area [136]. In our previous study, an alginate scaffold loaded with MSC exosomes was also developed to treat nerve injury-induced pain [48].

**Figure 7:**
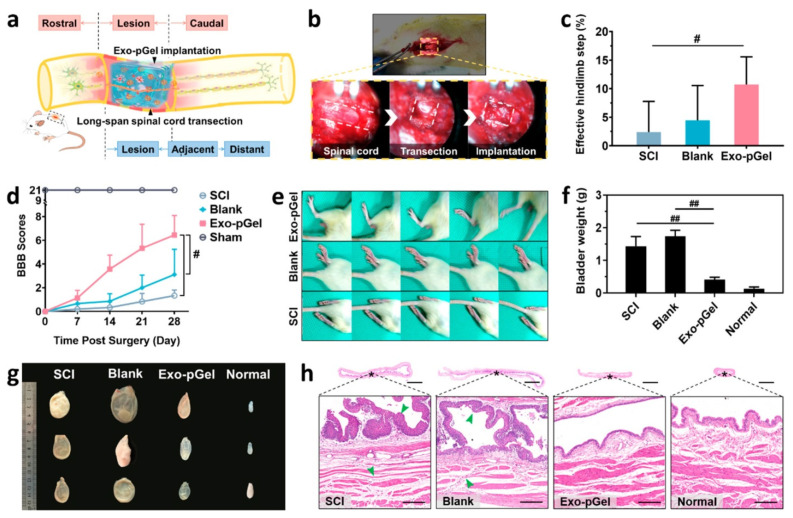
Effects of human mesenchymal stem cell exosomes loaded in peptide-modified adhesive hydrogel (Exo-pGel) on long-span spinal cord transection model. (**a**) Graphical representation of spinal cord injury (SCI) model with Exo-pGel treatment. (**b**) Surgical procedure for spinal cord transection and implantation. (**c**) Hind-limb ladder walking tests on Day 28. ^#^ *p* < 0.05 by Mann-Whitney U test. (**d**) Basso, Beattie, and Bresnahan (BBB) locomotor score. ^#^ *p* < 0.05 by Mann-Whitney U test. (**e**) Typical records of walking gaits on Day 28. (**f**) Weights of bladder on Day 28. ^##^ *p* < 0.01 by two-tailed unpaired *t*-test. (**g**) Morphological changes of bladders on Day 28. (**h**) Hematoxylin and eosin staining images of bladder tissue. The boxed images show the magnified views of the fields marked by the asterisks. Reprinted with permission from Li, L.; Zhang, Y.; Mu, J.; Chen, J.; Zhang, C.; Cao, H.; Gao, J. (2020). Copyright 2020 Nano Letters, American Chemical Society [138].

In a sciatic nerve defect model, a chitin conduit embedded with human gingiva MSC-derived exosomes were found to promote Schwann cell proliferation and axon growth from the dorsal root ganglion [137]. In addition, this scaffold increased the number and diameter of nerve fibers and enhanced myelin formation, nerve transmission, and motor function. In another SCI model, exosomes embedded within peptide-modified hydrogel stimulated nerve regeneration and preserved urinary function [138] (Figure 7). Recent studies on exosome scaffolds for nerve injury repair are summarized in Table 3.

## 9. Conclusions

Various biomaterials and fabrication techniques have been developed to construct 3D scaffolds suitable for the promotion of nerve injury repair. Natural polymeric materials are advantageous due to their inherent biocompatibility and biodegradability. However, rapid biodegradability can limit their applications. Many bio-scaffolds have been investigated for therapeutic efficacy using a wide array of nerve injury models. In general, the results show that these bio-scaffolds can provide neuroprotection, promote repair, decrease lesion volume, and improve functional recovery in animal models. In particular, bio-scaffolds with embedded multipotent MSCs have proven to be safe and effective in various CNS and PNS disease models. The microenvironment provided by these bio-scaffolds plays a major role in determining the stem cell lineage and ultimate regeneration success, so much effort has gone into the design and fabrication of ideal 3D biomaterials. Recently, bio-scaffolds have been developed that continuously release exosomes containing factors promoting regeneration, including neurotrophins, mRNAs, and miRNAs.

Though bio-scaffolds have many advantages such as high biocompatibility, cell adhesion/differentiation, and biodegradation ability, they have their own limitations, such as low mechanical stability, thermal sensitivity, rapid degradation, contamination risks, expensive cost of production, and complicated processing methodologies. In addition to the bio-scaffolds mentioned in this review, recently there are other novel inorganic nanomaterials such as phosphorene and borophene that are promising for nerve regeneration [140,141,142]. In summary, scaffolds with bioactive cells or an exosome hold greater promise for nerve injury treatment.

## Figures and Tables

**Table 1:** Recent in vitro studies using bio-scaffolds for nerve injury repair.

Bio-Scaffold	Cell Type	Disease	Results	Reference
PDGF-MS-containing tubular scaffold	Neural progenitor	Spinal cord injury	Promoted both growth and migration of MUSE-NPCs	[129]
3D collagen scaffold	Glioma	Glioma	Good biocompatibility with glioma cells and able to influence gene expression and biological functions	[130]
Scaffold incorporating salmon fibrin, HA, and laminin	Human neural stem cells	Neurovascular niche	Enhanced vasculogenesis from human endothelial colony-forming cell-derived endothelial cells for cellular therapeutics	[131]
Chitosan-based scaffold	Radial glia	Traumatic brain injury	Effective cellular and growth factor delivery vehicle for cell transplantation	[132]
Collagen scaffold	Neural stem cells	Spinal cord injury	Promoted nerve regeneration and locomotor function	[71]

Abbreviations: PDGF-MS: platelet-derived growth factor-microsphere; MUSE-NPCs: neural progenitor cells differentiated in vitro from multilineage-differentiating stress-enduring cells.

**Table 2:** Recent studies using bio-scaffolds for nerve injury repair in animal models.

Bio-Scaffold	Species	Disease	Results	Reference
Poly (propylene fumarate) polymer with collagen biomaterial	Rat	Spinal cord injury	Promoted neurotrophy, neuroprotection, myelination, and synapse formation, and reduced CSPG deposits and fibrotic scarring	[133]
3D collagen-based scaffold	Mouse	Neuroblastoma	Promoted microenvironment within scaffold and helps in cell transplantation and drug delivery	[134]
Collagen nerve conduit	Rat	Sciatic defect	Promoted motor nerve regeneration	[69]
Chitosan hydrogel scaffold	Mouse	Ischemic brain injury	Improved tissue regeneration following hind-limb ischemia	[51]
3D fibrin hydrogel scaffold	Rat	Spinal cord injury	Promoted aligned axonal regrowth and locomotor function	[105]
Collagen/heparin/VEGF scaffold	Rat	Traumatic brain injury	Provided an excellent microenvironment for nerve regeneration	[72]
Collagen scaffold	Rat	Spinal cord injury	Improved locomotor function and nerve regeneration	[71]
Silk fibroin scaffold	Rat	Traumatic brain injury	Neuroprotection	[97]
RADA16-BDNF self-assembling peptide hydrogel scaffold	Rat	Traumatic brain injury	Enhanced the growth, survival, and differentiation of MSCs by providing a favorable microenvironment	[135]
Chitin scaffold	Rat	Sciatic nerve injury	Improved sciatic nerve regeneration, myelin sheath formation, and functional recovery	[64]
Keratin sponge	Rat	Sciatic nerve injury	Regulated inflammatory cytokine release from macrophages, axon extension, and nerve regeneration	[109]
Fibrin hydrogel	Rat	Sciatic nerve defect	Promoted regeneration as well as the secretion and signaling of multiple neurotrophic factors	[106]
Keratin sponge	Rat	Spinal cord injury	Improved functional recovery and inhibition of inflammatory response through macrophage polarization	[110]

Abbreviations: CSPG: chondroitin sulfate proteoglycans; VEGF: vascular endothelial growth factor; BDNF: brain-derived neurotrophic factor; MSCs: mesenchymal stem cells.

**Table 3:** Recent examples of exosome scaffold use in nerve injury models.

Bio-Scaffold	Exosome Source	Disease	Results	Reference
Peptide-modified adhesive hydrogel	Human MSC-derived	Spinal cord injury	Promoted nerve regeneration and protected urinary tissue by easing oxidative stress and inflammation	[138]
Alginate scaffold	Human umbilical cord MSC-derived	Nerve injury-induced pain	Anti-nociceptive, anti-inflammatory, and neurotrophic effects	[48]
Chitin conduit	Human gingiva MSC-derived	Rat sciatic nerve defect	Increased the number and diameter of nerve fibers and promoted myelin formation	[137]
Chitosan hydrogel	Human placental MSC-derived	Hind-limb ischemia	Enhanced angiogenesis and tissue regeneration	[51]
Pituitary adenylate cyclase-activating polypeptide 38	Retinal ganglion cell (RGC)-derived	Traumatic optic neuropathy	Promoted retinal ganglion cell survival and axon regeneration	[139]

## Data Availability

Not applicable.
